# Global transcriptome profiling of wild soybean (*Glycine soja*) roots under NaHCO_3 _treatment

**DOI:** 10.1186/1471-2229-10-153

**Published:** 2010-07-26

**Authors:** Ying Ge, Yong Li, Yan-Ming Zhu, Xi Bai, De-Kang Lv, Dianjing Guo, Wei Ji, Hua Cai

**Affiliations:** 1 Plant Bioengineering Laboratory, The College of Life Sciences, Northeast Agricultural University, Harbin, China; 2 State Key Lab for Agrobiotechnology and Department of Biology, The Chinese University of Hong Kong, Shatin, N.T., Hong Kong

## Abstract

**Background:**

Plant roots are the primary site of perception and injury for saline-alkaline stress. The current knowledge of saline-alkaline stress transcriptome is mostly focused on saline (NaCl) stress and only limited information on alkaline (NaHCO_3_) stress is available.

**Results:**

Using Affymetrix^® ^Soybean GeneChip^®^, we conducted transcriptional profiling on *Glycine soja *roots subjected to 50 mmol/L NaHCO_3 _treatment. In a total of 7088 probe sets, 3307 were up-regulated and 5720 were down-regulated at various time points. The number of significantly stress regulated genes increased dramatically after 3 h stress treatment and peaked at 6 h. GO enrichment test revealed that most of the differentially expressed genes were involved in signal transduction, energy, transcription, secondary metabolism, transporter, disease and defence response. We also detected 11 microRNAs regulated by NaHCO_3 _stress.

**Conclusions:**

This is the first comprehensive wild soybean root transcriptome analysis under alkaline stress. These analyses have identified an inventory of genes with altered expression regulated by alkaline stress. The data extend the current understanding of wild soybean alkali stress response by providing a set of robustly selected, differentially expressed genes for further investigation.

## Background

Soil salinity-alkalinity is one of the major environmental challenges limiting crop productivity globally. For example, the western Songnen Plain of China, which has 3.73 million ha of sodic land, is one of the three major contiguous sodic soil regions in the world. Understanding the molecular basis of plant response under saline-alkaline conditions will facilitate biotechnology efforts to breed crop plants with enhanced tolerance to high saline-alkaline. Root is an important organ for carrying water and mineral nutrients to the rest of the plant. As the primary site of perception and injury for salinity and alkaline stress, roots provide an ideal target for study of the molecular mechanism underlying plant saline-alkaline stress tolerance and adaptation [1].

Soybean is rich in nutraceutical compounds, e.g., isoflavone and saponins. Its high symbiotic nitrogen fixing capacity (100 Kg/ha/year; FAO data 1984) helps to replenish soil nitrogen. Therefore, soybean is an ideal crop for crop rotation and intercropping. Wild soybean exhibits much higher adaptability to suboptimal (i.e. stressful) natural environment compared to the cultivated soybean. The wild soybean (*Glycine soja*) line used in this study can germinate and set seed in the sodic soil at pH9.02 and survive in the nutrient solution with 50 mmol/L NaHCO_3_. The physiological stress response of wild soybean has been described previously [2]. The obvious advantage of wild soybean over other extremophile model plants is that it can be directly compared with soybean cultivar to generate useful information for elucidation of plant stress tolerance and adaptation.

High throughput technologies, such as microarray, have been used to examine the gene expression patterns under various environmental cues in *Arabidopsis *[1,3-5], rice [6], wheat [7,8], grape [9] and soybean [10]. Although studies on plant sodic stress has been conducted in perennial plant *Leymus chinensis *[11], *Puccinellia tenuiora *[12,13], *Limonium bicolor *[14] and *Tamarix hispida *[15] using cDNA array, the dynamic expression change under sodic stress is not yet available. Currently, commercialization microarrays are only available for a small number of species. Therefore, hybridization using a microarray for a closely related species was used and has demonstrated feasible, without discernible loss of information [16]. Ji has demonstrated that feasible to investigate the wild soybean's gene expression profile using the Affymetrix^® ^Soybean Genome Genechip^® ^based on the high similarity between the two allied species by comparison between the EST sequences of *Glycine soja *and *Glycine max *[17].

In the present study, we analyzed the transcriptome changes in *Glycine soja *roots under NaHCO_3 _treatments using Affymetrix^® ^Soybean Genome Array. Our objectives were threefold: (1) to identify genes regulated by alkaline stress, (2) to identify genes co-regulated in a similar pattern and their dynamic change over the course of stress treatment, and (3) to identify the expression feature of gene family and their function category.

## Results and Discussion

### Transcriptome profiling data

The data discussed in this publication have been deposited in NCBI's Gene Expression Omnibus [18] and are accessible through GEO Series accession number GSE17883.

The assessment of duplicated microarray experiments using correlation analysis was shown in Additional file 1. The Pearson coefficients ranged from 0.953 to 0.993. A total of 23849 probe sets were considered Present, among which 23741 showing consistent expression patterns in the replicates (0 < median (SD/mean) < 0.5) were used in the following analysis.

### Validation of microarray data by real-time quantitative PCR

**Figure 1:**
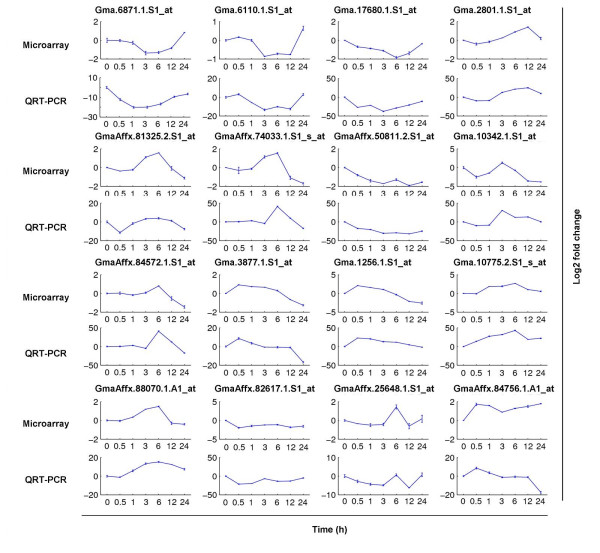
**Validation of microarray expression with QRT-PCR**. Genes were randomly selected from our findings. The x-axis represents hours of stress. The y-axis is log2 fold change.

The real-time quantitative PCR (QRT-PCR) expressions of 16 candidate genes relative to a reference gene, gapdh, were compared with microarray expression in Figure 1. Although the magnitude of the transcript abundance varied between the microarray and QRT-PCR, the patterns were similar. This indicates that the cross-species hybridization obtains real expression values of *Glycine soja*'s genes.

### Identification of genes differentially expressed throughout the NaHCO_3 _stress

**Figure 2:**
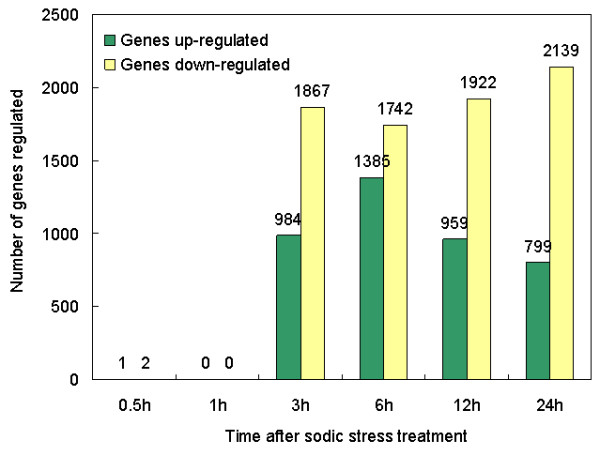
**Number of genes differentially expressed in roots under saline-alkaline stress treatment**. Total number of genes differentially up- (green bars) and down-regulated (yellow bars) in roots under 50 mmol/L NaHCO_3 _stress treatment compared with the sample without stress (P < 0.05, q < 0.15). The x-axis represents hours of stress. The y-axis represents number of probe sets. The tissues for RNA extraction were harvested at the indicated time points. See Methods for data normalization, processing, statistical analysis and classification of differentially expressed genes.

A total of 7088 probe sets were differentially expressed in roots under saline-alkaline stress (P < 0.05, q < 0.15) (Additional file 2) and their distribution at each time point was illustrated in Figure 2. Comparisons of the kinetics of changes in expression patterns revealed four features. First, 2851, 3127, 2881 and 2938 probe sets were significantly differentially expressed (up/down-regulated) under NaHCO_3 _stress at 3, 6, 12 and 24 h, respectively. The number of differentially expressed genes ranged from 3.37 to 9.01% of total genes. The number of significantly differentially expressed genes peaked at 6 h and decreased at 12 and 24 h. This result is the same as the pattern found in *Puccinellia tenuiflor *[12] and *Limonium bicolor *[14] that the frequency of genes showing differential expression at 6 h is higher than that at 12, 24 and 48 h. These suggest that the gene expressions change considerably after 6 h alkaline stress. In addition, it is noteworthy that the total number of differentially expressed genes and the dynamic patterns of up-regulated genes are similar to *Arabidopsis *transcriptome profile under salt stress but differed from that under cold, drought or osmotic stimuli [3]. This further suggests that plants have distinctive mechanisms in coping with ionic and physical stress. Second, genes up-regulated showed a trough at 6 h whilst, to a different extent, genes down-regulated showed a peak at 6 h. This indicates a biphasic response of this organ to alkaline stress, and 6 h is the turning point. Third, almost no observable changes of transcripts were detected until after 3 h stress treatment. We found that it takes three hours for the roots to respond to sodic stress at transcription level. This might attribute to the timescale of molecular concentration and modifications (e.g. intake of Na^+ ^and HCO_3_^-^, transcription of mRNA). Cellular response to extracellular signals was reported to be a time-sequential process [19,20]. The process is most likely distributed over hours and requires the involvement of both protein signaling and gene regulation. Processes including changes of mRNA concentrations, protein complex formation, translocation in space, conformational changes or their molecular modifications, occur on different timescales ranging from milliseconds to seconds (e.g. induced conformational changes) via seconds and minutes (e.g. post-translational protein modification) up to hours and days for gene expression kinetics and/or even years by epigenetic regulation. Fourth, 3307 probe sets were up-regulated and 5720 were down-regulated. Moreover, the number of down-regulated genes was greater than that of the up-regulated genes at each time point, which is contrary to the *Arabidopsis *profile under abiotic stresses [3]. We presume that this may attribute to the concomitant Na^+^, high pH, CO_3_^2- ^and HCO_3_^- ^in the NaHCO_3 _solution. Under multiple stress condition, plants suffer more severe injury than that of a single stress.

**Figure 3:**
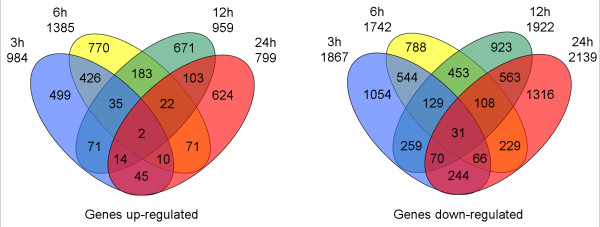
**Venn diagram, depicting the overlap of regulated genes at various time points**. The number outside the circle denotes the total number of genes up- (on the left) or down- (on the right) regulated at specific time point. The number within one circle or more than two circles denotes the time specific genes or overlapped genes, respectively.

The Venn diagram (Figure 3) showed that the number of specifically up-regulated genes ranged from 50.71 to 78.10% of total genes at each time point. Only two probe sets were constantly up-regulated and 31 were constantly down-regulated at 3 h, 6 h, 12 h and 24 h. One of the two up-regulated probe sets (Gma.56.1.S1 at) encodes a putative phosphoenolpyruvate carboxylase kinase (pepck2), which is nodule-enhanced and regulated throughout nodule development [21]. This protein also increases in response to increasing intracellular pH in *Arabidopsis *[22]. The other one (Gma.13972.2.S1 at) encodes an uncharacterised protein containing regulator of chromosome condensation (RCC1) domain. The RCC1 protein is known to be involved in the maintenance of chromatin conformation, in regulation of chromosome condensation, and in monitoring and signalling of DNA replication [23]. The constant up regulation of this gene, which is similar to *Arabidopsis *subjected to cold and oxidative stresses [3], indicates that adjustment of chromosome structure is needed under stress condition. The number of specifically down-regulated genes ranged from 45.24 to 61.52% of total genes at various time points.

### Co-expression analysis of stress regulated genes

Time course analysis using a method described by Storey [24] revealed 1592 probe sets (q < 0.001) displaying significantly changed expression (Additional file 3). Hierarchical clustering of averaged expression value from two biological replicates identified 8 distinctive patterns for the 1592 genes (Additional file 4).

**Figure 4:**
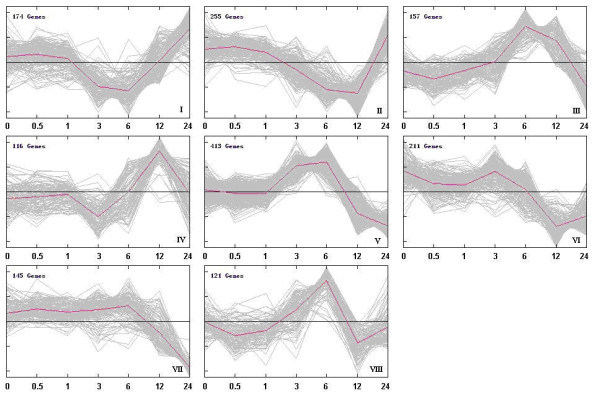
**Dynamic expression pattern of different clusters during NaHCO_3 _stress**. Genes with altered expression over time were identified by Edge [80,81] time course methodology (q value < 0.001). K-means clustering was performed to identify 8 clusters, each containing various numbers of genes with similar expression pattern under NaHCO_3 _stress. The red lines show representative transcriptional regulators. The x-axis represents the stress treatment time in hours. The y-axis represents normalized log2 microarray expression data.

K-means clustering was conducted to analyze the co-expression setting and 8 clusters with coordinated expression patterns were identified (Figure 4). These clusters reflected the general trends and key transitional states during NaHCO_3 _stress. Gene lists for each cluster, including normalized expression values, can be found in additional file 5. Clusters I, II and IV contain genes down-regulated after 1 h, but up-regulated at different time points. Differ from the other two Clusters; genes in Cluster IV were down-regulated after 12 h. Clusters III, V and VIII contain genes up-regulated after 1 h, but down-regulated after 6 h. Genes in Cluster VIII were up-regulated after 12 h and genes in cluster VII exhibited a more dramatic decrease after 6 h. In cluster VI, genes exhibited a minor decrease after 3 h.

### Functional categorization and pathway of differentially expressed genes

**Figure 5:**
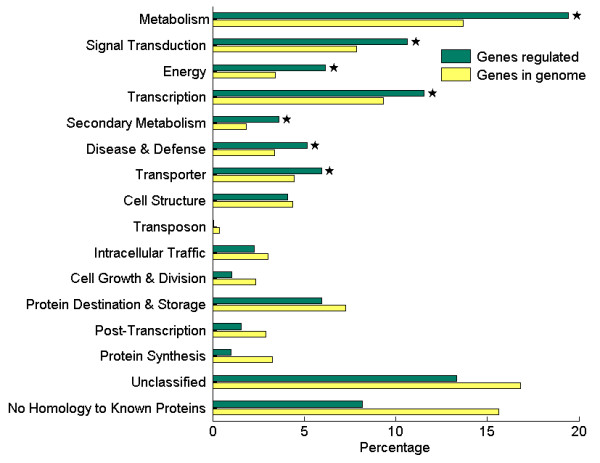
**Functional categorization of genes respond to NaHCO_3 _stress and of all genes in *Glycine soja***. There are 16 functional categories. Functional categorization for gene group regulated by NaHCO_3 _stress treatment (total number, 1592) (green bars) and for whole genome genes (yellow bars) were shown with their percentages. Category significantly over-represented in the respond group was shown with asterisk (P < 0.01, FDR < 0.05).

Out of the 1592 significantly changed probe sets, we assigned at least one GO term to 1380 probe sets based on sequence similarity. The distribution of functional categories is showed in Figure 5. As seen, 7 GO over-represented (P < 0.01, FDR < 0.05) categories were: metabolism signal transduction, energy, transcription, secondary metabolism, transporter, disease and defence. These biological processes were affected by the sodic stress treatment.

**Figure 6:**
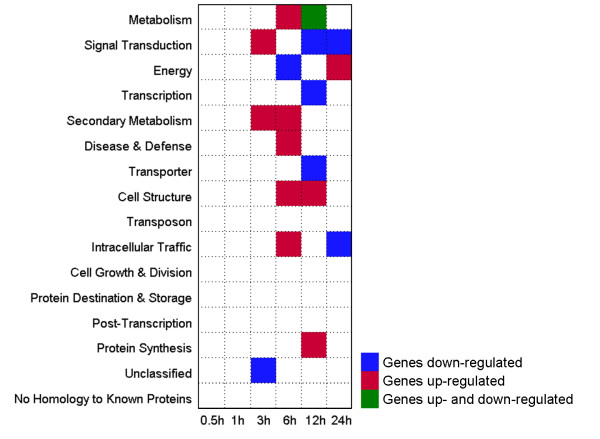
**GO enrichment analysis of the differentially expressed genes**. Blue or Red square denotes the functional category over-represented in up-or down-regulated genes at that time point. Green square denotes the functional category over-represented in both up-and down-regulated genes. The x-axis represents the length of stress treatment time. The y-axis represents functional categories (p < 0.01, FDR < 0.05).

**Figure 7:**
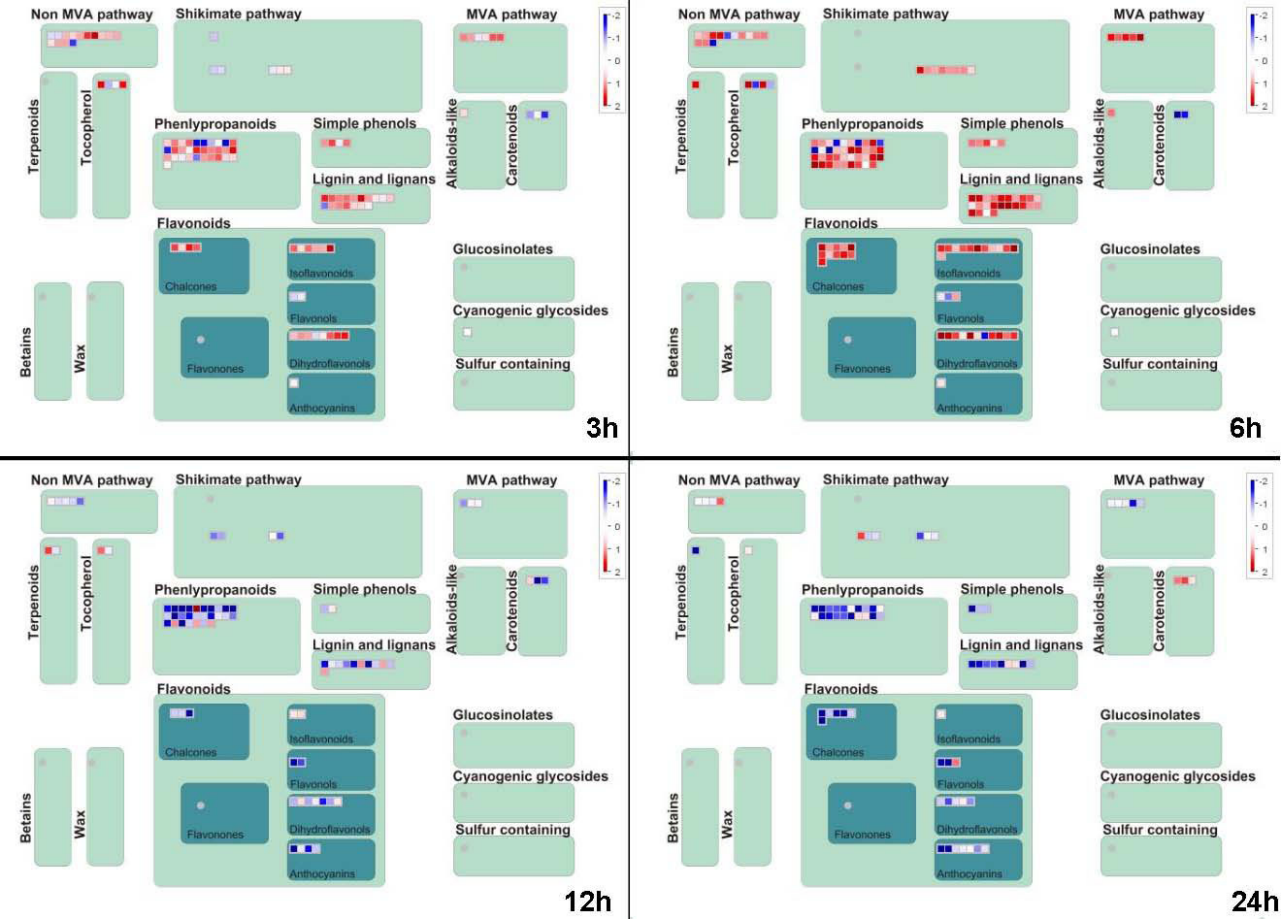
**Genes up/down regulated in the overview of secondary metabolism**. Mapman was used to visualize the secondary metabolism pathways with genes up/down regulated at 3 h, 6 h, 12 h, and 24 h. In the display, each BIN or subBIN is represented as a block where each transcript is displayed as a square, which is either colored red if this transcript is up- or blue if this transcript is down-regulated.

To understand the relationship between response time points of transcripts and their biology meanings, GO enrichment analysis (P < 0.05, q < 0.15) and MapMan [25,26] visualization were conducted. As illustrated in Figure 6, genes participate in signal transduction and secondary metabolisms were over-represented at earlier time points (3 h). These secondary metabolisms related genes, which significantly participate in phenylpropanoid, simple phenols, flavonoids, lignin and lignans, and non-mevalonate (MVA) pathway, were presented up-regulated under sodic stress (Figure 7). Phenylpropanoid metabolism was found induced under stress (including salt) [27,28]. Similar to the signal transduction process, these secondary metabolisms might generate a cascade response for their early induction after stress treatment.

Genes involved in metabolism, secondary metabolism, disease and defence, cell structure, intracellular traffic were induced after 6 h and genes involved in energy production were decreases after 6 h. Furthermore, genes responsible for signal transduction, transcription and transporter were decreased at later time point (12 h). Signal transduction and intracellular traffic were decreased at 24 h, while energy was induced. These observations were further supported by a more specific comparison of metabolism using MapMan. This analysis showed up-regulation of several biosynthetic pathways at 6 h, such as terpenes, flavonoids, phenylpropanoids & phenolics, TCA, sucrose metabolism, lignin and lignans and non-mevalonate (MVA) pathway (Additional file 6A). The number of genes participate in secondary metabolism were found more at 6 h than that at 3 h (Figure 7). A further investigation was done to the JA synthesis (Additional file 6B). It was observed that lipoxygenases and oxophytodienoate reductase were up-regulated at 6 h, indicating that the JA synthesis pathway participates in the early response to sodic stress. Several biosynthetic pathways were down-regulated at 12 h, such as cell wall modification, flavonoids, phenylpropanoids & phenolics and lipids metabolism pathways (Additional file 6A). A custom MapMan pathway image was generated and AP2-EREBP, WRKY, bZIP, MYB and MYB related, C2C2 and C2C2-CO-like transcript factors were decreased at 12 h (Additional file 6C and 6D). Protein synthesis were induced after 12 h, and most of them were plastidic, misc and proteins in the nucleotide (Additional file 6E).

The above analysis revealed a cascade process: 1) Firstly, signal transduction and secondary metabolism were induced at 3 h; 2) As a result, metabolism, disease defense, cell structure and intracellular traffic were induced at 6 h; 3) After that, signal transduction, transcription and transporter decreased after 12 h; 4) Later on, signal transduction, secondary metabolism and intracellular traffic was induced at 6 h, and decreased at 24 h; 5) After a long period of stress treatment, protein synthesis and energy were induced at 12 h and 24 h, respectively.

Detailed descriptions of genes participate in signal transduction and transcription are as follows:

### Signal transduction

Approximately, 122 probe sets representing various signalling proteins, such as 14-3-3, protein phosphatase, small GTPases, and protein kinases, calmodulin-binding family proteins, were up-regulated at 3 h and down-regulated after 12 h. Stress tolerance or susceptibility in plants is a coordinated action of various genes including those signalling pathway components [29-31]. As expected, protein phosphatase and protein kinase were over-represented at earlier time points because reversible protein phosphorylation is a central mechanism in cellular signal transduction and transcriptional regulation [32]. Calmodulin-binding family protein, such as calcium-dependent protein kinase or calmodulin-like domain protein kinases (CDPKs) are essential sensor-transducers of calcium signalling pathways in plants [33]. Their up-regulation at the early stage endorsed the trigger of downstream components to cope with the stressful condition.

It is noteworthy that members of the 14-3-3 family protein were also up-regulated at the early stage of NaHCO_3 _stress. 14-3-3 family proteins, for its specific phosphoserine/phosphothreonine-binding activity [34], are thought to be involved in a large range of abiotic signalling processes and to interact with many regulatory proteins like transcription factors, plasma membrane H^+^-ATPase, ion channels, ascorbate peroxidase (APX) and abscisic acid (ABA) [35-38].

### Transcription factors

**Figure 8:**
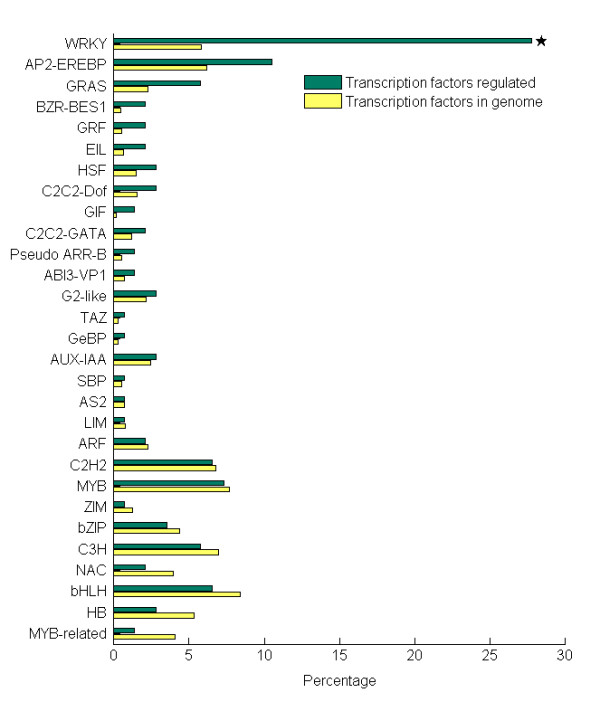
**Transcription factors families of the up-regulated genes at the early stage of NaHCO_3 _treatment**. There are 29 transcription factors families. Transcription factors families for gene group induced at 3 h and 6 h of NaHCO_3 _stress treatment (total number, 147) (green bars) and for whole genome genes (yellow bars) were shown with their percentages. Transcription factors family significantly over-represented in the respond group was shown with asterisk (P < 0.01, FDR < 0.05).

As above, two processes, transcription and transport, emerged at earlier time points. Probe sets up-regulated before 6 h were listed in Additional file 7. According to the putative annotation assigned by homology search with genes in *Arabidopsis *Gene Regulatory Information Server [39,40], 147 transcription factors representing 29 different families were found to be induced at earlier stage of stress treatment (3 h and 6 h). These transcription factor families were compared (Figure 8) to identify families playing main roles at earlier stress response.

Plant WRKY transcription factor superfamily are known to be involved in biotic [41] and abiotic stress [4] response, and in developmental processes [42]. However, their roles in mediating plant alkaline stress response are largely unknown. Recently, 64 *GmWRKY *genes were identified from soybean [43], and 30 probe sets representing 15 WRKY family members were quickly induced at early time point before decreasing at later time points. This pattern was similar to WRKYs expression pattern in response to other biotic or abiotic stresses in numerous plant species [42].

Similar to the WRKY superfamily, AP2-EREBP family are well known for their important functions in plant growth and development, especially in hormonal regulation and in environmental stress response [43,44]. Our results showed that transcripts encoding AP2-EREBP family proteins increased drastically after 3 h, and rapidly decreased after 12 h of stress treatment.

Although a member of GRAS family proteins seems to be involved in development and other processes, such as rhizobial Nod factor-induce [45], SHORT-ROOT movement[46], GA3 induction [47] and drought stress [48], very little is known about their physiological roles under saline or alkaline stress. The stress modulated expression of GRAS genes suggested they may be important in NaHCO_3 _stress response. A full list of GRAS family proteins in soybean still needs to be identified systematically.

In addition, the BZR1 and BES1 protein regulate subsets of BR-responsive genes as downstream signalling components [49] and are considered to mediate responses to other stimuli as well. The ethylene-insensitive3-like (EIL) transcription factor, which participates in ethylene signalling pathway [50], was also induced at the early stage of NaHCO_3 _stress treatment.

14-3-3 proteins are known to regulate several cellular processes and therefore are called as General Regulatory Factors (GRFs) [51]. We found that *GRF *family genes were up-regulated from 0.5 to 6 h, and decreased after 12 h. Recent investigation of 14-3-3 gene expression profile showed that they are also regulated by salt stress [52-54] and alkaline stress [11,12].

### MicroRNAs

Several stress-specific microRNAs have been identified in plants under various abiotic stresses, including nutrient deficiency [55,56], drought [57,58], cold [59], high salinity [58,60,61], UV-B radiation [62] and mechanical stress [63]. Some microRNA targets are stress-related genes, suggesting that microRNAs play important roles in plant stress response [64].

**Figure 9:**
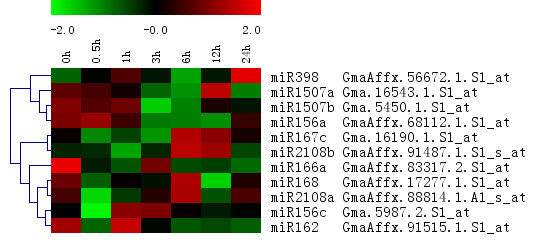
**Expression profile of pre-microRNAs under NaHCO_3 _stress**. The pre-microRNAs probe sets were annotated with the predicted microRNAs. Pearson correlation Hierarchical clustering of averaged expression value from two biological replicates was shown.

A BLASTn [65] search against 78 pre-microRNAs in miRBase (Release 13.0, March 2009) [66-69] identified 30 microRNAs in the microarray, among which 11 were called Present and were modulated by NaHCO_3 _stress. Hierarchical clustering using the average expression value of these 11 microRNAs identified 4 distinctive patterns as illustrated in Figure 9. MiR398, miR1507a, miR1507b and miR156a were reduced after 3 h and increased after 12 h. Mir398 targets two Cu/Zn superoxide dismutases (CSD1 and CSD2) and was reported to decrease dramatically under oxidative stress in *Arabidopsis *[70]. MiR156a was increased under salt stress in *Arabidopsis*, and targets 2 SBP family transcription factors which play essential role in vegetative phase change and root development [59]. MiR1507a and miR1507b were known to be involved in nitrogen fixation in soybean nitrogen-fixing nodules [71]. Similar to the observation in *Arabidopsis *[59], MiR167c and miR2108b were significantly up-regulated at 6 h. MiR166a, miR168 and miR2108a form a cluster with distinctive dynamic pattern (decreased after 0.5 h, followed by an increase after 3 h before a decrease after 6 h). MiR168 has been reported to be salt stress regulated and target to ARGONAUTE1, which is related to plant development [59,72]. The stress modulated miRNAs suggest their possible roles in alkaline stress.

## Conclusions

This is the first comprehensive transcriptome profiling analysis of wild soybean root under alkaline stress. The current knowledge about plant alkaline stress response is limited and we provide a list of genes showing dynamic expression change under NaHCO_3 _stress. Functional characterization of these genes highlights the common and distinctive mechanisms underlying plant response to alkaline and other abiotic stress. Most of the alkaline-modulated genes are involved in metabolism, energy, signal transduction and transcription. Some molecular processes, such as signal transduction, secondary metabolism, and regulation of transcription, were induced at earlier time points. Genes involved in these processes accomplished their regulatory mission and decreased after 12 h. As a result, protein synthesis and energy metabolism were induced. These data indicate that the cellular pathways respond to the NaHCO_3 _stress as a cascade process.

## Methods

### Plant material, growth conditions, and stress treatments

*Glycine soja *L. seeds were grown in a culture room with the following settings: 60% relative humidity, 24°C and a light regime of 16 h light/8 h dark. The light source SON-T ARGO 400 W generated constant illumination of 30000 lx. Before sowing, seeds of *Glycine soja *L. G07256 were shaken for 10 min in 98% sulfuric acid. Subsequently, seeds were washed five times with sterile water. Thirty seeds were placed on each petri dish to accelerate germination for 2 days. Germinated seedlings were then transferred into the growth boxes containing 1/4 strength Hoagland's solution. Nineteen days after sowing, seedlings in the stress treatment group were transferred into 1/4 strength Hoagland's solution with 50 mmol/L NaHCO_3 _(pH 8.5) before exposure to light condition for 3 h.

### Tissue harvest and RNA isolation

Roots from 3 cm root apex were harvested in two independent biological replicates after 0, 0.5, 1, 3, 6, 12 and 24 h treatment with 50 mmol/L NaHCO_3 _stress under the same light condition. Samples were immediately frozen in liquid nitrogen, and stored at -80°C. To minimize biological variance, roots from three plants originating from the same experiment, condition and cultivar were pooled, and the extracted RNA was used for microarray hybridization. Total RNA was extracted from frozen roots with TRIzol (Invitrogen, Carlsbad, CA) according to the instructions from the manufacturer. RNA integrity was evaluated on agarose gels electrophoresis and absorbance 260/280 ratios between 1.8 and 2.2 were typically obtained.

For QRT-PCR experiments, reverse transcription was carried out using the SuperScript^® ^III First-Strand Synthesis System (SKU# 18080-051, Invitrogen) according to the manufacturer's instructions. Prior to the QRT-PCR assays, the quality of the cDNA was assessed by PCR with gapdh-specific primers to test for genomic DNA contamination.

### DNA chip hybridization

GeneChip^® ^Soybean Genome Array (Cat. # 900526; Affymetrix^®^; Santa Clara, CA, USA) containing 37,744 *Glycine max *probe sets (35,611 transcripts) was used for microarray analysis. This high-density array consists of 11-probe pair (25 bp per oligonucleotide) and provides multiple independent measurements for each individual transcript. cDNA labelling and Affymetrix^® ^hybridization was carried out by Gene Tech Biotechnology Company Limited (Shanghai, China) according to a Affymetrix^® ^protocol (Affymetrix^®^, Santa Clara, CA) outlined in [73].

### Microarray Data Analysis

The computation of expression values were conducted using dChip software [74] (Cheng Li Lab, Harvard). We adopted a sample wise normalization to the median probe cell intensity (CEL) of all 14 arrays. For each sample, the median CEL intensity of one replicate was scaled to the median CEL intensity of all arrays and defined as baseline. The remaining replicates of each sample were normalized to the baseline applying an Invariant Set Normalization Method [75]. Model-based gene expression was obtained from normalized CEL intensities based on a Perfect Match-only model [75]. The quality of each repeated experiment was tested by performing a Pearson's Correlation of signal intensities. Present/Absent/Marginal calls were generated from scanned arrays using Affymetrix^® ^GCOS 1.4 software. Only genes present at least in one of the two biological replicates of each time point were considered as Present [76].

Two types of analysis were conducted to identify differentially expressed genes. First, two-sample t-test was used to evaluate differential expression of genes between each time point (P < 0.05) [77]. The data were further filtered based on the False Discovery Rate (FDR, q value < 0.15) [78,79]. Second, Edge [80,81] time course methodology was used to test for genes with changed expression changes over time (q value < 0.001). Hour was chosen for class variable and covariate giving time points; Differential Expression Type was Time course; Spline type was Natural cubic spline.

Pearson correlation Hierarchical Clustering and K-Means Clustering were performed with TM4: MeV 4.3 [82,83]. Details of the GeneChip^® ^soybean genome array are available at the Affymetrix^® ^website [84]. The annotation and functional categories for these transcripts were assigned based on the Soybean GeneChip^® ^annotation file (Updated Oct. 2007) and *Arabidopsis *ATH1 array annotation file (Updated Sept. 2007) [85]. To assess the significance of over-represented GO terms or the transcription factor families in the list of the regulated genes against the genome, Fisher's Exact Test (p < 0.01) [86] and Benjamini and Hochberg method (FDR < 0.05) [79] were used. The visualization of profiling data sets in the context of existing knowledge (pathway) was performed with MapMan [23,24]. The mapping file is Gmax_AFFY_09 (1.0).

### Real-time quantitative PCR

The glyceraldehyde-3-phosphate dehydrogenase (gapdh, AFFX-r2-Gma-gapdh-M_at, accession # DQ355800) was used to normalize all values in the QRT-PCR assays, because it exhibited the lowest variation in expression values throughout the NaHCO_3 _treatment (average fold change = 1.096, coefficient of variation = 0.114). Primers for QRT-PCR were designed using Primer3 software [87]. Primer sequences were listed in Additional file 8.

QRT-PCR reactions based on SYBR Green fluorescence were performed using SYBR GreenER™ using qPCR SuperMix Universal (SKU# 11762-500, Invitrogen) on a Bio-rad iQ5 Real-Time PCR Detection System with iQ™5 Optical System Software Version 2.0 (BIO-RAD, HERCULES, CA, USA) following the manufacturer's instructions. One microliter of synthesized cDNA (diluted 1:10) was used as template. The preset cycling parameters for a SYBR Green experiment with a dissociation curve were used. The analysis term settings were set at an amplification-based threshold, an adaptive baseline, and a moving average. The amplification efficiencies were determined by analyzing the standard curves generated from triplicate series of five cDNA template dilutions. The iQ™ 5 Optical System Software Version 2.0 plotted the known starting quantities against the measured Ct values and generate the standard curve. The amplification reactions were consisted of a 2-min denaturing step at 95°C, followed by 40 cycles at 95°C for 10 s, 60°C for 30 s and 70°C for 30 s, end with melting curve program 70°C for 30 s. Three replicate reactions per sample were used to ensure statistical significance. The RNA from each sample was analyzed simultaneously. Expression levels for all candidate genes were computed based on the stable expression level of the reference gene according to Pfaffl method [88].

## Authors' contributions

YG conceived of the study, participated in its design, carried out the material preparation, microarray data analysis and drafted the manuscript, participated in QRT-PCR. YL conceived of the study, participated in its design and assisted with statistical analysis. XB participated in its design and coordination. DKL assisted with the program composition and pre-microRNA prediction. WJ participated in QRT-PCR. HC participated in its coordination. YMZ and DJG conceived of the study, participated in its design and coordination, and helped with the manuscript editing. All authors read and approved the final manuscript.

## Supplementary Material

Additional file 1 **Assessment of the replicated experiments using Pearson's correlation analysis**. X and Y-axis represent the two replicates at each time point. The colour of each square denotes the Pearson's correlation coefficient of the two experiments as indicated in the legend. Click here for file

Additional file 2 **Differentially expressed genes at each time point after NaHCO_3 _stress**. Total number of genes differentially up- and down-regulated in roots under 50 mmol/L NaHCO_3 _stress treatment compared with the sample without stress (P < 0.05, q < 0.15). The Probe sets were annotated based on the Soybean GeneChip^® ^annotation file (Updated Oct. 2007). The fold value was counted using the average expression value between the sample after stress and control. Click here for file

Additional file 3 **Differentially expressed genes throughout the NaHCO_3 _stress period**. Time course analysis revealed 1592 probe sets (q < 0.001) displaying significantly changed expression. Rank was ordered by Q-value. Click here for file

Additional file 4 **Hierarchical cluster analysis of genes differential expressed throughout the NaHCO_3 _stress period**. Pearson correlation Hierarchical clustering of averaged expression value from two biological replicates was shown. Click here for file

Additional file 5 **List of genes in each cluster from K-means clustering**. Pearson correlation K-Means Clustering of averaged expression value from two biological replicates was shown. Click here for file

Additional file 6 **Pathway visualized with MapMan**. Pathways up/down regulated at 3 h, 6 h, 12 h, and 24 h were shown. Click here for file

Additional file 7 **Probe sets up-regulated at certain time point before 6 h NaHCO_3 _stress**. Probe sets significantly up-regulated at 0.5 h, 1 h, 3 h or 6 h under 50 mmol/L NaHCO_3 _stress treatment were listed (P < 0.05, q < 0.15). Click here for file

Additional file 8 **Genes and primer sequences used in the QRT-PCR assays**. Genes, their primer sequences and their sodic stress response patterns were listed. Click here for file
