# Supplementary material for: Global transcriptome profiling of wild soybean (Glycine soja) roots under NaHCO3 treatment
Source: BMC Plant Biol. 2010 Jul 26;10:153. doi: 10.1186/1471-2229-10-153 (PMC3017823; doi:10.1186/1471-2229-10-153)
Supplement: Additional file 1 — Assessment of the replicated experiments using Pearson's correlation analysis. X and Y-axis represent the two replicates at each time point. The colour of each square denotes the Pearson's correlation coefficient of the two experiments as indicated in the legend. [file 1471-2229-10-153-S1.DOC]

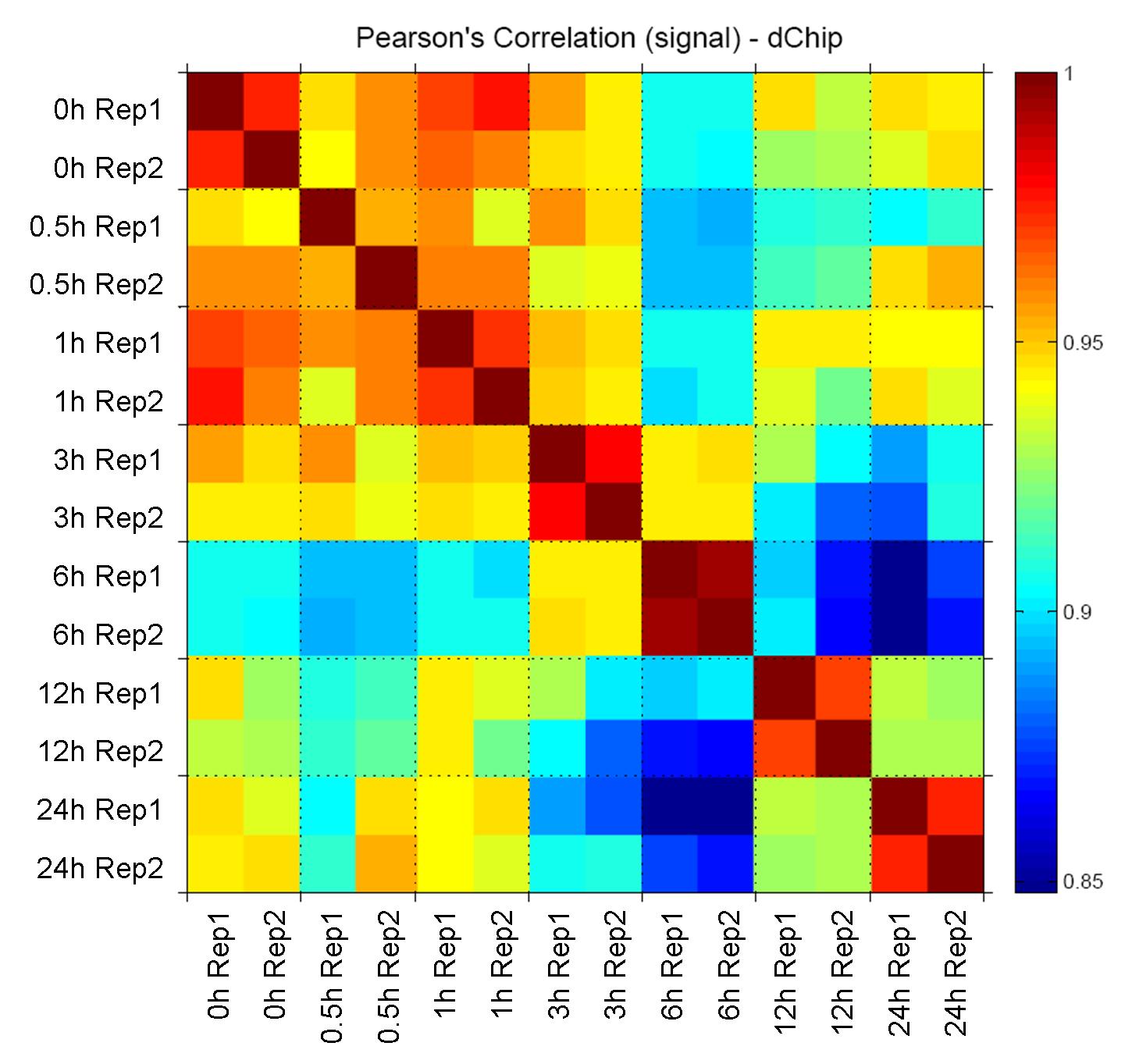


Additional file 1 --- Assessment of the replicated experiments using Pearson's correlation analysis.

X and Y-axis represent the two replicates at each time point. The color of each square denotes the Pearson's correlation coefficient of the two experiments as indicated in the legend.
