# Supplementary material for: Global transcriptome profiling of wild soybean (Glycine soja) roots under NaHCO3 treatment
Source: BMC Plant Biol. 2010 Jul 26;10:153. doi: 10.1186/1471-2229-10-153 (PMC3017823; doi:10.1186/1471-2229-10-153)
Supplement: Additional file 6 — Pathway visualized with MapMan. Pathways up/down regulated at 3 h, 6 h, 12 h, and 24 h were shown. [file 1471-2229-10-153-S6.PPT]

## Slide 1
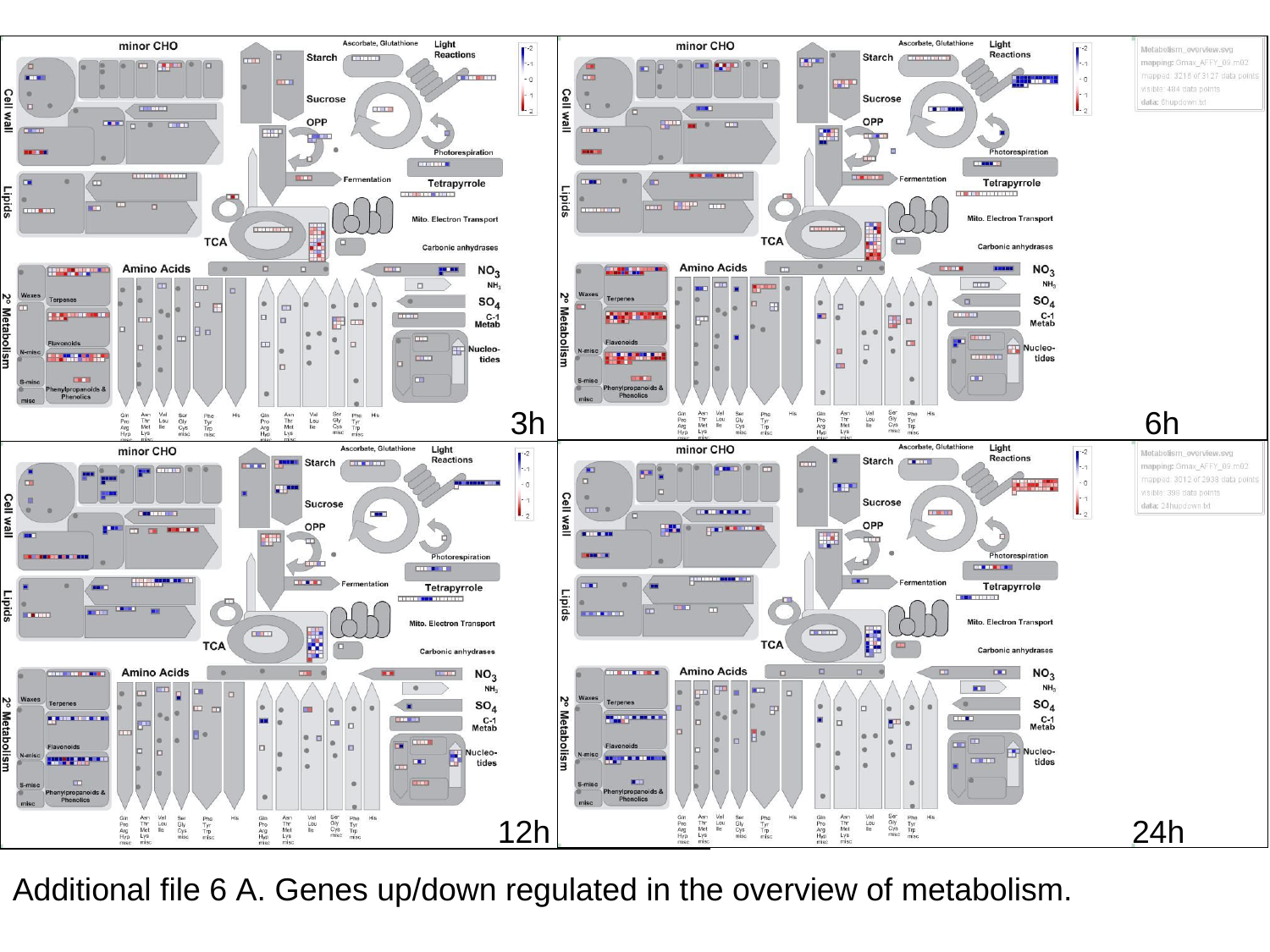

#
3h
6h
12h
24h
Additional file 6 A. Genes up/down regulated in the overview of metabolism.

## Slide 2
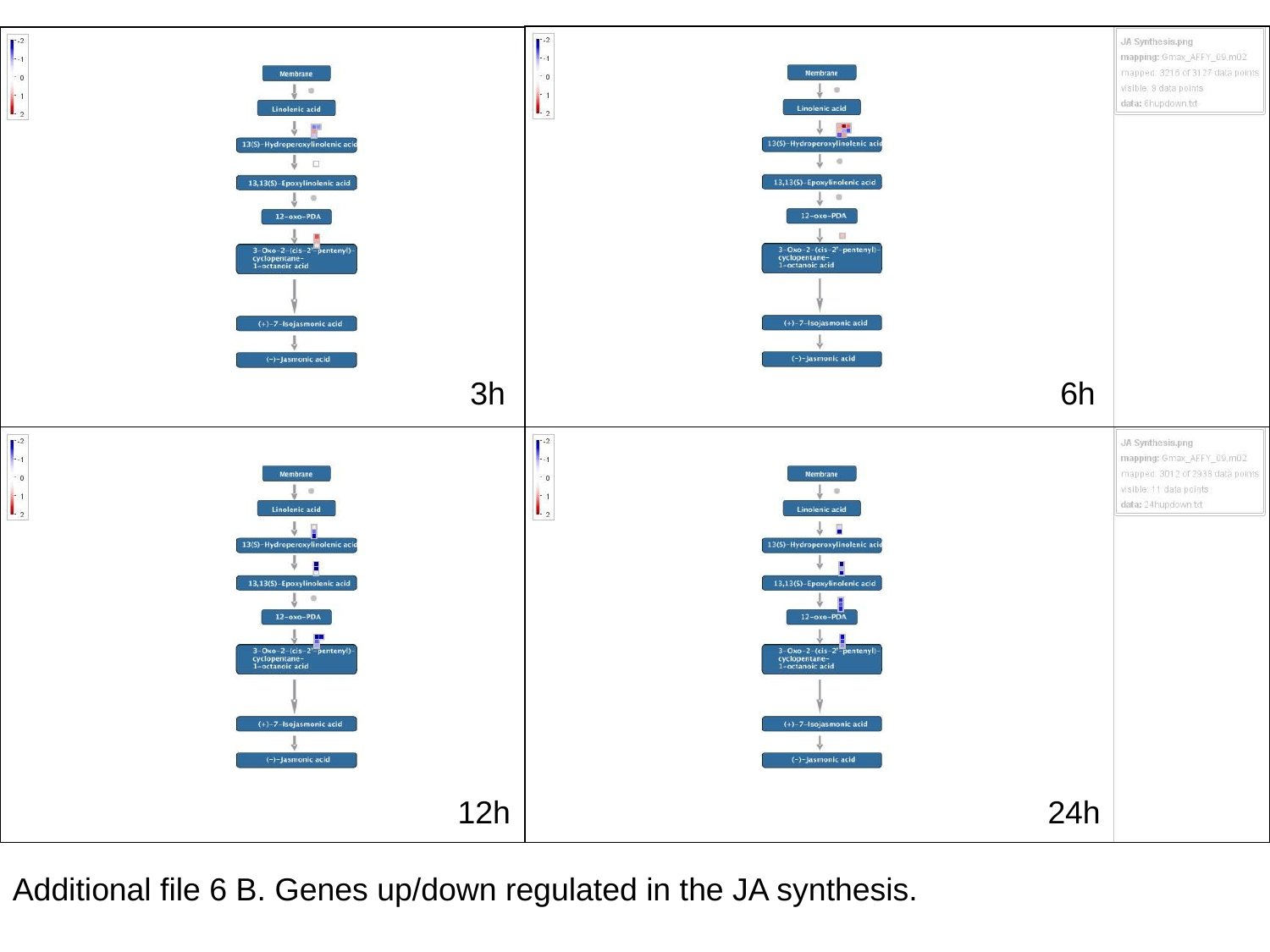

#
3h
6h
12h
24h
Additional file 6 B. Genes up/down regulated in the JA synthesis.

## Slide 3
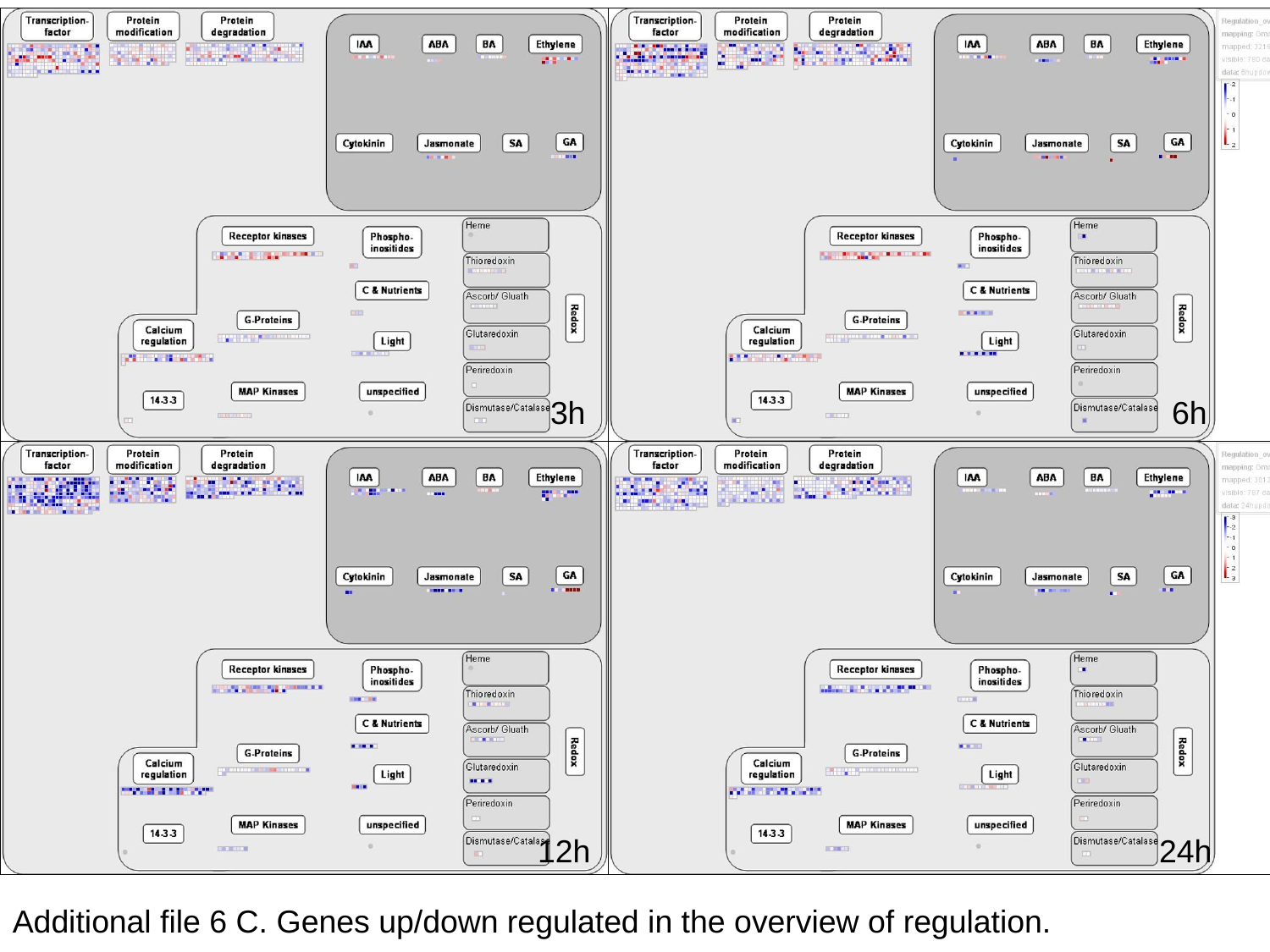

#
3h
6h
12h
24h
Additional file 6 C. Genes up/down regulated in the overview of regulation.

## Slide 4
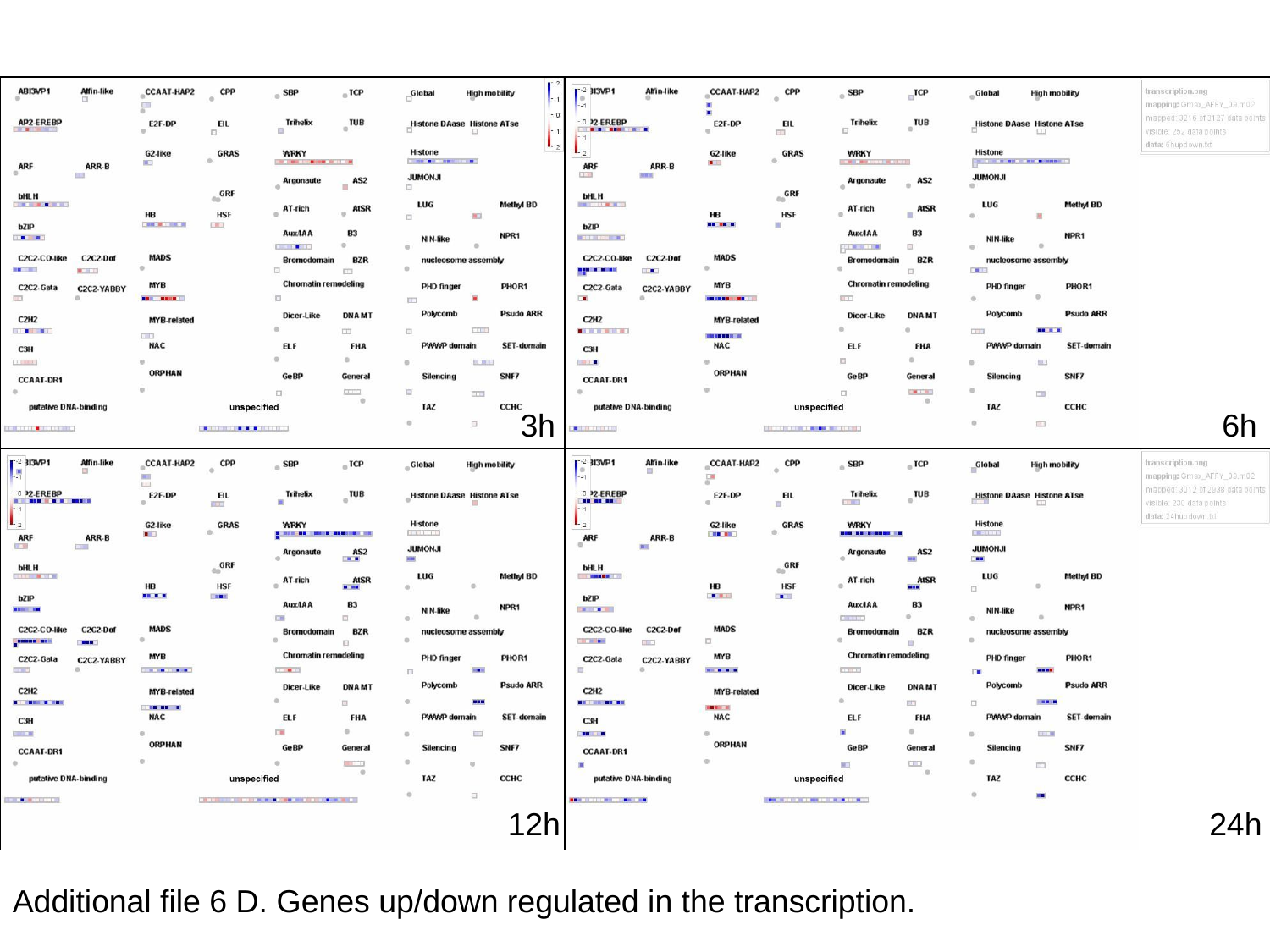

#
3h
6h
12h
24h
Additional file 6 D. Genes up/down regulated in the transcription.

## Slide 5
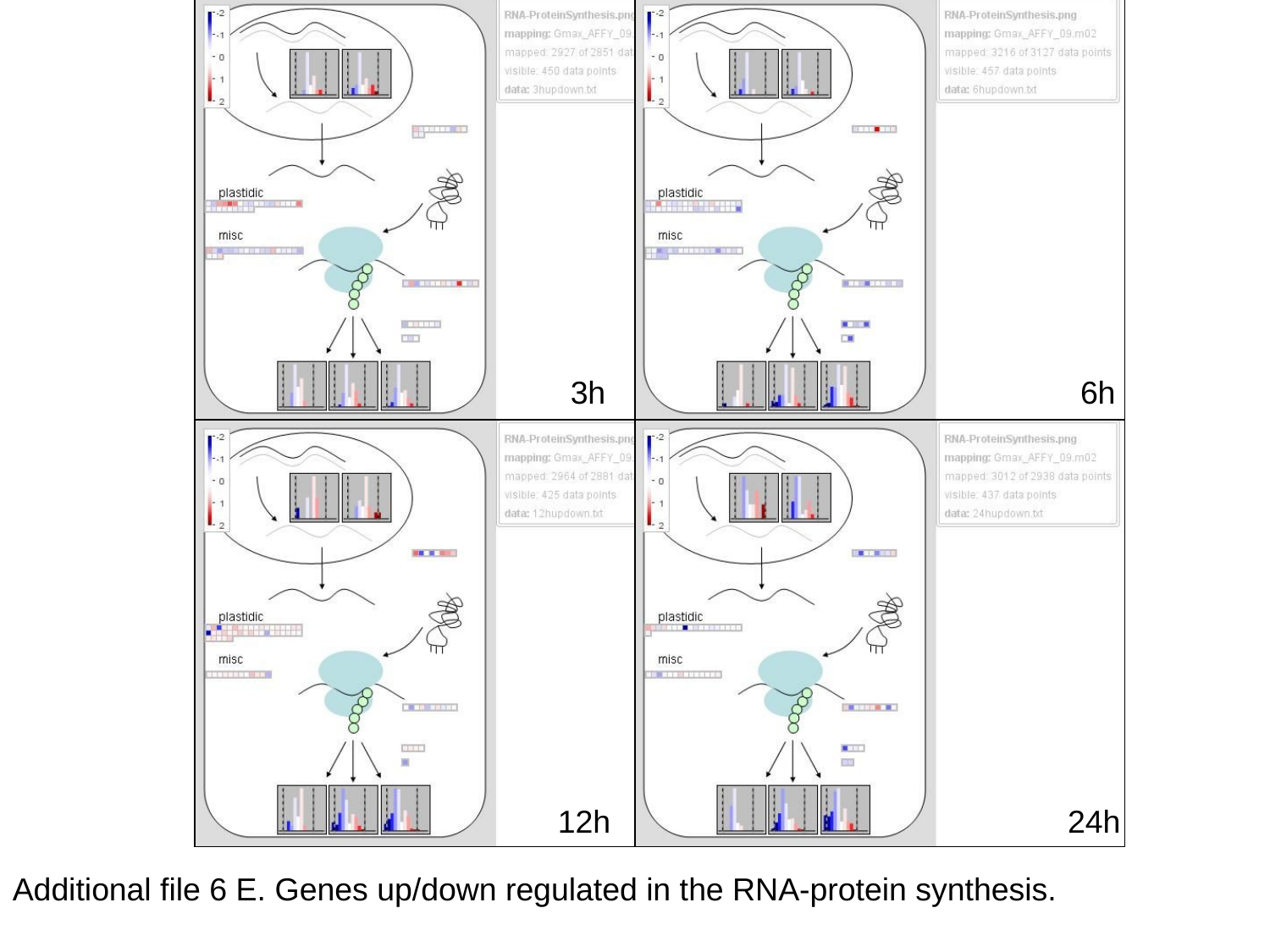

#
3h
6h
12h
24h
Additional file 6 E. Genes up/down regulated in the RNA-protein synthesis.
